# Atomic Structure and Biochemical Characterization of an RNA Endonuclease in the N Terminus of Andes Virus L Protein

**DOI:** 10.1371/journal.ppat.1005635

**Published:** 2016-06-14

**Authors:** Yaiza Fernández-García, Juan Reguera, Carola Busch, Gregor Witte, Oliberto Sánchez-Ramos, Christian Betzel, Stephen Cusack, Stephan Günther, Sophia Reindl

**Affiliations:** 1 Department of Virology, Bernhard-Nocht-Institute for Tropical Medicine, Hamburg, Germany; 2 Department of Pharmacology, University of Concepción, Concepción, Chile; 3 European Molecular Biology Laboratory, Grenoble Outstation, Grenoble, France; 4 Gene Center, Department of Biochemistry, Ludwig-Maximilians-Universität, Munich, Germany; 5 Laboratory for Structural Biology of Infection and Inflammation, University of Hamburg, Hamburg, Germany; Institut Pasteur, FRANCE

## Abstract

Andes virus (ANDV) is a human-pathogenic hantavirus. Hantaviruses presumably initiate their mRNA synthesis by using cap structures derived from host cell mRNAs, a mechanism called cap-snatching. A signature for a cap-snatching endonuclease is present in the N terminus of hantavirus L proteins. In this study, we aimed to solve the atomic structure of the ANDV endonuclease and characterize its biochemical features. However, the wild-type protein was refractory to expression in *Escherichia coli*, presumably due to toxic enzyme activity. To circumvent this problem, we introduced attenuating mutations in the domain that were previously shown to enhance L protein expression in mammalian cells. Using this approach, 13 mutant proteins encompassing ANDV L protein residues 1–200 were successfully expressed and purified. Protein stability and nuclease activity of the mutants was analyzed and the crystal structure of one mutant was solved to a resolution of 2.4 Å. Shape in solution was determined by small angle X-ray scattering. The ANDV endonuclease showed structural similarities to related enzymes of orthobunya-, arena-, and orthomyxoviruses, but also differences such as elongated shape and positively charged patches surrounding the active site. The enzyme was dependent on manganese, which is bound to the active site, most efficiently cleaved single-stranded RNA substrates, did not cleave DNA, and could be inhibited by known endonuclease inhibitors. The atomic structure in conjunction with stability and activity data for the 13 mutant enzymes facilitated inference of structure–function relationships in the protein. In conclusion, we solved the structure of a hantavirus cap-snatching endonuclease, elucidated its catalytic properties, and present a highly active mutant form, which allows for inhibitor screening.

## Author Summary

Hantaviruses may cause severe disease in humans, either hemorrhagic fever or cardiopulmonary syndrome. Both conditions are associated with high lethality. Vaccines and effective treatments approved for application in humans are not available. The L protein plays a central role in hantavirus replication and represents a key target for antiviral drug development. However, the protein is difficult to study, as it can hardly be expressed in cells, and therefore structure and function are largely unknown. We succeeded to express and purify the N-terminal domain of ANDV, a South American hantavirus, in mutant forms. The atomic structure of the domain was solved by X-ray crystallography. The structural data in conjunction with our enzymatic activity data demonstrate that the N-terminal part of ANDV L protein harbors an endonuclease that cleaves RNA, presumably to “steal” cap structures from cellular mRNA for virus transcription. The investigation of a range of mutant enzymes revealed the detailed role of amino acid residues within the endonuclease. Importantly, using a known inhibitor of viral endonucleases we demonstrate the potential of the ANDV enzyme as an antiviral target. The discovery of a highly active mutant form of the endonuclease, which can be expressed at high level, will facilitate drug discovery programs for hantaviruses.

## Introduction

Andes virus (ANDV) belongs to the genus *Hantavirus* within the family *Bunyaviridae*. Hantaviruses can be pathogenic to humans and are distributed worldwide [1–3]. Their natural hosts are various rodent species [4]. ANDV is endemic in Argentina and Chile and its main reservoir host is the long-tailed pygmy rice rat (*Oligoryzomys longicaudatus*) [5]. The virus causes hantavirus cardiopulmonary syndrome (HCPS) that is associated with a case fatality of up to 40%. Related HCPS-causing hantaviruses are found throughout the Americas. However, in contrast to other hantaviruses, ANDV can be transmitted from human to human [6, 7].

The hantavirus genome consists of three RNA segments: L (large), M (medium), and S (small) [8]. The L segment encodes the 250-kDa L protein, which contains an RNA-dependent RNA polymerase (RdRp) domain in the center as indicated by sequence homology [9]. The L protein is likely to possess additional enzymatic functions involved in transcription and replication of the RNA genome. The M segment contains the gene for the glycoprotein precursor, which is cleaved co-translationally into the envelope proteins Gn and Gc. The S segment encodes the nucleoprotein, which encapsidates the viral genome. Several hantaviruses encode a second gene product in the S segment, the non-structural protein NSs. This small protein seems to play a role in modulating the immune response of the host cell [10, 11].

The 5’ ends of hantavirus mRNAs contain a stretch of heterologous nucleotides suggesting that the virus uses a mechanism called ‘cap-snatching’ to initiate transcription of its mRNAs [12, 13]. Cap-snatching involves two steps. First, the 5’ end of a cellular mRNA is bound by a viral cap-binding protein and secondly, the cap is cleaved off several nucleotides downstream by a viral endonuclease. The capped RNA fragment is used as a primer to initiate transcription of the viral mRNA by the RdRp. The viral strategy of stealing the 5’ cap from host cell mRNA was first described for influenza A virus (IAV; family *Orthomyxoviridae*) [14]. Here, the cap-binding and endonuclease domains reside in the PB2 and PA proteins, respectively [15–19]. In other segmented negative strand RNA viruses, the endonuclease resides in the very N terminus of the L protein, as shown for La Crosse virus (LACV; genus *Orthobunyavirus*, family *Bunyaviridae*), Lassa virus, and lymphocytic choriomeningitis virus (both family *Arenaviridae*) [20–23]. An endonuclease motif (H-PD-D/E-K) is also found in the N terminus of hantavirus L proteins, although there is no experimental evidence for a functional enzyme. The endonuclease is a potential target for antiviral therapy, as cap-snatching is an essential step in virus replication [24–35].

Investigation of L protein of ANDV and other hantaviruses has been challenging, because the protein is difficult to express in heterologous systems [36, 37]. We have previously shown that this phenotype is determined at post-transcriptional level and that mutations in the putative endonuclease can rescue L protein expression in mammalian cells [37]. Both suggests that a strong catalytic activity of the ANDV endonuclease prevents high-level expression by down regulating the level of its own and host cell mRNAs.

The aim of the current study was to provide proof for the existence of a cap-snatching endonuclease in ANDV L protein using a structural and biochemical approach. We took advantage of the previously characterized mutations enhancing the L protein level in mammalian cells to facilitate expression of the endonuclease domain in bacteria.

## Results

### Expression and purification of ANDV endonuclease

For structural and biochemical characterization of the putative endonuclease of ANDV, we aimed to express the N terminus of L protein in *E*. *coli*. To this end, plasmids for expression of the N-terminal 163, 179, 191, 194, 197, 200, 211, 214, and 228 amino acid residues of wild-type ANDV L protein were generated using *E*. *coli* cloning strain DH5α. To facilitate purification, the expression constructs contained an N-terminal His-tag or His-tag plus maltose-binding protein (MBP), respectively. However, we encountered difficulties in expressing the L protein fragments in bacteria, as observed previously in mammalian cells [36, 37]. Proteins with less than 200 residues were found exclusively in inclusion bodies. Transformation of expression strains of *E*. *coli* with constructs containing 200 or more residues failed repeatedly, suggesting that background expression of soluble wild-type endonuclease is already toxic to the cells. To circumvent this problem, we took advantage of 15 amino acid exchanges that enhanced L protein expression in mammalian cells, presumably because they attenuate the enzymatic activity of the endonuclease [37]. All 15 mutations were individually introduced into the plasmids for expression of L protein amino acid residues 1–200 fused to an N-terminal His-tag (ANDV L_1–200_) and the mutated constructs were tested for expression in *E*. *coli* BL21 cells. The results are summarized in Table 1. Two mutants (L_1–200_ Y32V and D37A) showed a phenotype like the wild-type protein, i.e. no bacterial growth was observed. The remaining 13 mutants (L_1–200_ R35H, H36R, D40E, I43A, K44A, N50A, P96A, D97E, N98A, E110A, K124A, K127A, and N167A) were successfully expressed with varying yield and purified via nickel affinity and size exclusion chromatography (S1 Fig). Noticeable slower growth of expressing *E*. *coli* was observed for L_1–200_ N167A. A thermofluor assay [38] was used to evaluate the stability of the mutant proteins under various conditions. The ANDV L_1–200_ mutants were most stable at high salt concentrations and low pH (S2 Fig), as expected from their calculated isoelectric point of approximately 7.5. Therefore, we used pH 5.5 and 1 M NaCl for protein purification. All mutants eluted from the size exclusion column according to their molecular mass, indicating they form monomers in solution.

**Table 1:** Summary of structural and functional data for ADNV endonuclease mutants and hypothetical role of mutated residues.

Mutation	ANDV L_full_	ANDV L_1–200_			
	Expression in mammalian cells^1^	Expression in bacteria^2^	Thermal stability^3^	Stabilization by Mn^2+^ ^4^	Ribonuclease activity^5^
*Wild-type*					
None	–	–	NA	NA	NA
*Active site residues involved in catalysis and Mn* ^*2+*^ *coordination*					
H36R	+++	++	43°C	+ 5°C	0
D97E	+++	+++	47°C	–	0
E110A	+++	++	45°C	+ 7°C	0
*Active site residues positioning catalytic residues or substrate*					
D37A	+	–	NA	NA	NA
P96A	+++	+++	45°C	+ 10°C	14
K124A	+++	++	49°C	+ 12°C	2
K127A	++	++	46°C	+ 12°C	15
*Residues contributing to substrate binding outside the active site*					
Y32V	++	–	NA	NA	NA
R35H	+++	+++	45°C	+ 11°C	20
*Residues with stabilizing contacts near the active site*					
D40E	+++	+	37°C	+ 11°C	96
I43A	+++	+	34°C	+ 10°C	83
K44A	+++	+	38°C	+ 13°C	75
N50A	+	+++	41°C	+ 12°C	22
N98A	+	+	43°C	+ 11°C	83
*Residue positioning helix e*					
N167A	+	+	38°C	+ 13°C	100

^1^ ANDV full-length L protein expression in mammalian cells upon transient transfection. The steady-state protein level was estimated from immunoblot signals shown by Heinemann et al. [37].

^2^ Semiquantitative representation of protein yield from + to +++. A “–”denotes apparent toxicity of the expressed protein preventing growth of transformed bacteria. A reduced growth rate was observed with bacteria expressing L_1–200_ N167A protein.

^3^ Melting temperatures measured by thermofluor assay.

^4^ Increase in melting temperature upon the addition of 16 mM MnCl_2_ measured by thermofluor assay.

^5^ Measured by ribonuclease assay. The percentage of degraded substrate RNA after 2 h of incubation is given in %.

NA, not applicable.

### ANDV endonuclease structure

**Fig 1:**
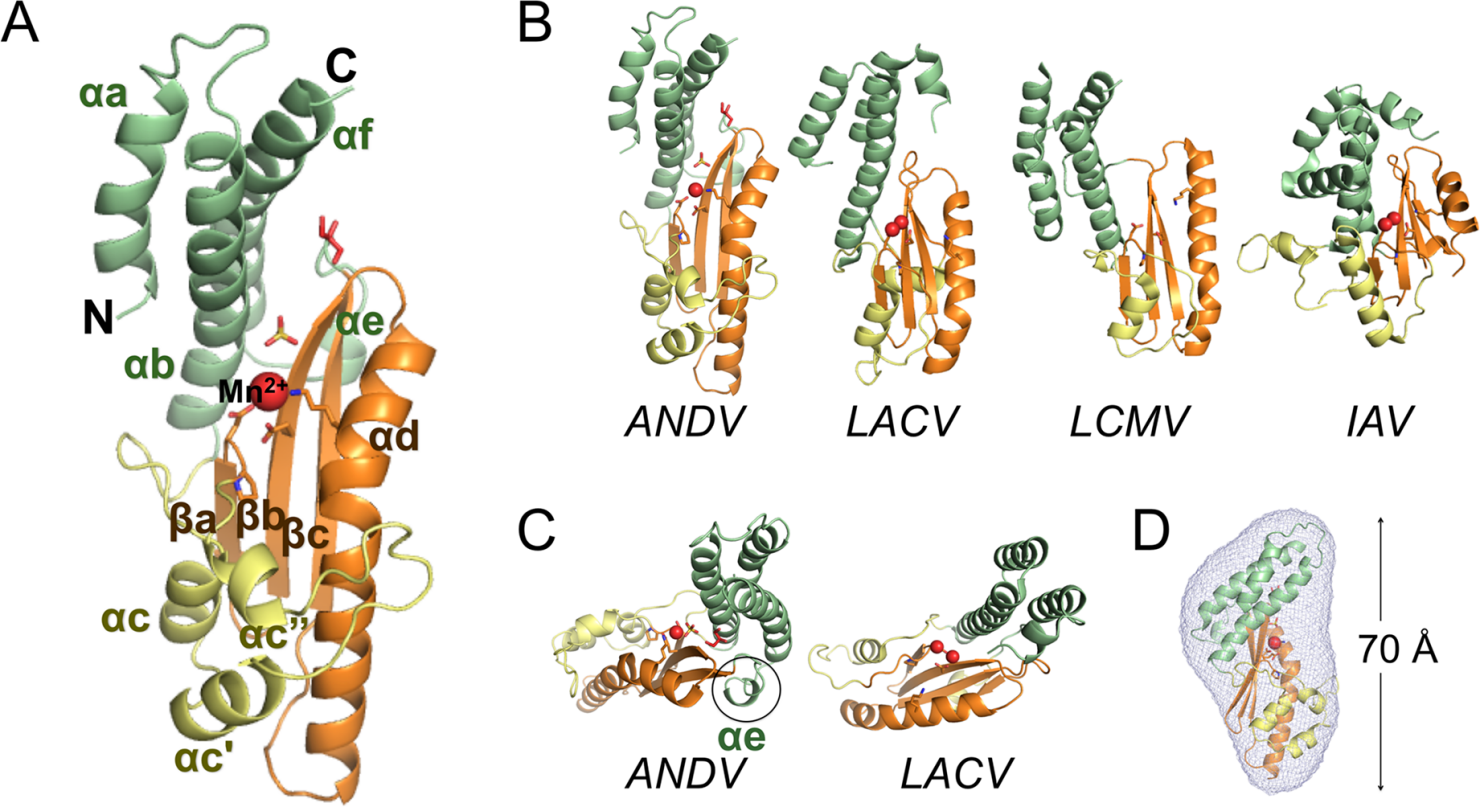
Atomic structure of ANDV L_1–200_ K127A and comparison with related cap-snatching endonucleases. **A**, Ribbon diagram of the ANDV L_1–200_ K127A structure. N and C termini are marked and secondary structure elements are annotated in analogy to related published structures. The conserved structural core is colored in orange, the conserved helix-bundle domain in green, and the remaining helical part in yellow. The manganese ion is shown as red sphere. Side chains of the active site residues and bound sulfate and glycerol molecules are shown as sticks. **B**, Comparison of ANDV L_1–200_ K127A with the cap-snatching endonucleases of orthobunyavirus LACV (PDB ID 2XI7), arenavirus LCMV (PDB ID 3JSB), and orthomyxovirus IAV (PDB ID 2W69). Structural elements are colored as in A. **C**, Top view of ANDV endonuclease showing the presence of a small helix αe (encircled) compared to the LACV structure, where this element is lacking. **D**, Superimposition of SAXS derived molecular shape with crystal structure (ribbon diagram) confirms the elongated and flat shape of the ANDV endonuclease.

ANDV L_1–200_ K127A was chosen for structural studies, as this mutant was the most stable mutant, which retained the capacity to bind to manganese ions—a co-factor of nucleases—along with a residual enzymatic activity (for details, see section on thermal stability below). The mutant was expressed as His-tag fusion of variable lengths (200, 211, 214, and 228 L protein residues) and subjected to crystallization trials. We succeeded to crystallize ANDV L_1–200_ K127A in the presence of Mn^2+^ after cleavage of the His-tag. The crystal structure was solved in space group P42_1_2 by molecular replacement using coordinates for residues 32–162 from the endonuclease of Hantaan virus ([39], co-submission), allowing for the building of a contiguous polypeptide chain for one molecule in the asymmetric unit and refinement to a resolution of 2.4 Å (see S1 Table, 5HSB.pdb). Clear electron density was visible for the complete structure with the exception of the loop between the first two α-helices (residues 13–20), where the signal was weak. The structure clearly shows a Mn^2+^ ion bound to the active site (Fig 1A), as verified by an anomalous signal from data collected at 1.77 Å.

The crystal structure of ANDV L_1–200_ K127A and the comparison to the cap-snatching endonucleases of LACV, LCMV, and IAV [15, 19, 21–23] provides clear evidence for the N terminus of ANDV L protein being an endonuclease (Fig 1B). ANDV L_1–200_ has an overall structure similar to the other viral endonucleases with the highest similarity to its closest relative LACV. Considering the almost lacking sequence homology between the four proteins—except the catalytic H-PD-D/E-K motif—the structural homology is surprisingly high: RMSD values between the structural core (orange) of ANDV endonuclease and LACV, LCMV and IAV endonucleases are 1.7 Å, 1.9 Å and 2.2 Å, respectively (as given by DALI [40]. The central β-sheet with the long α-helix αd that runs parallel to the β-sheet forms the core of the protein and provides the crucial active site residues in all four endonucleases (Fig 1A and 1B, shown in orange). Further structural elements are helical, although number, length, as well as position of the α-helices vary significantly between the structures. In all endonucleases, the active site is located in a groove between two lobes. One lobe is formed by a helix bundle that consists of two to three α-helices from the N terminus and at least one long α-helix (starting around residue 160) from the C-terminal end of the domain (Fig 1A and 1B, shown in green). The interaction between the last residues of the C-terminal helix and the first residues of the second helix stabilizes the 3-helix bundle (αa, αb, αf) and provides a plausible explanation why constructs with less than 200 residues were misfolded and thus insoluble. The other lobe contains less conserved structural elements consisting of two to three small helices (Fig 1A and 1B, shown in yellow). ANDV features a short additional α-helix αe between strand βc and the C-terminal helix αf, which is not present in LACV (Fig 1C).

One of the obvious differences between the endonuclease of ANDV and the other viruses are the overall dimensions of the domain: ANDV L_1–200_ is long and flat with approximate outer dimensions of 70 Å × 45 Å × 30 Å, whereas the other structures are more compact, in particular that of the distantly related IAV. The outer dimensions of the protein in the crystal fit with the dimensions in solution as determined by small angle X-ray scattering, indicating that the overall shape was not affected by crystallization artifacts (Figs 1D and S3).

### Surface charge distribution of ANDV endonuclease

**Fig 2:**
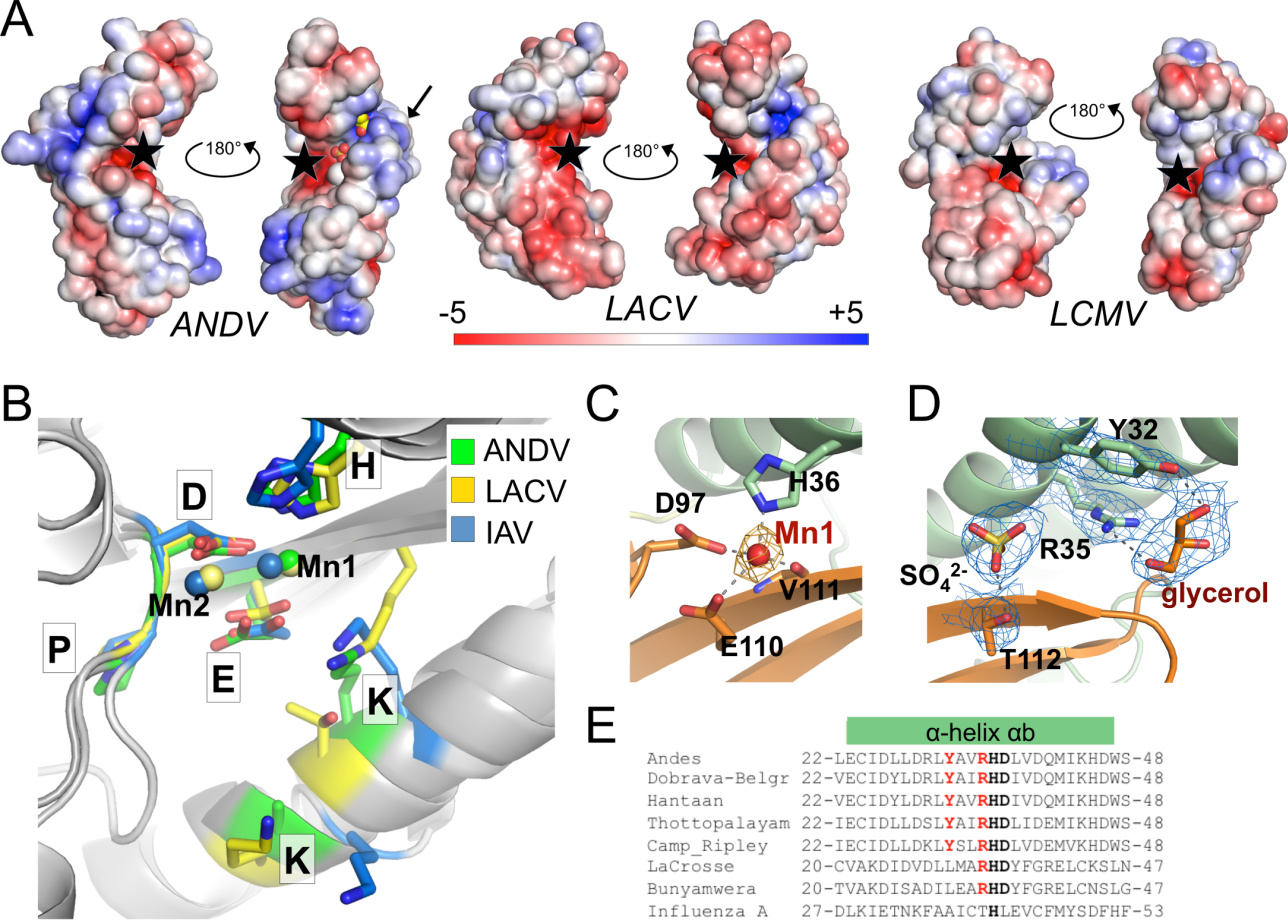
Surface charge distribution and active site arrangement of ANDV endonuclease compared to other cap-snatching endonucleases. **A**, Electrostatic surface potential of the endonucleases of ANDV, LACV, and LCMV. The active site is marked with a black star, the location of helix αe in the ANDV structure is marked with a black arrow, sulfate and glycerol molecules bound in the basic groove of ANDV endonuclease are shown as spheres. The surface potential is shown from -5 KT/e in red to +5 KT/e in blue. **B**, Superimposition of the side chains of active site residues and the Mn^2+^ ions of ANDV, LACV, and IAV endonucleases. **C,** Manganese coordination in the active site of ANDV endonuclease. Metal coordinating residues are shown as sticks and coordination with dotted lines. The anomalous difference Fourier map for the manganese atom is shown as orange mesh at 3σ. **D,** Electron density for a sulfate and a glycerol molecule close to the active site of the ANDV endonuclease (2|Fo|-|Fc| map at 2σ is shown as blue mesh; ligands and coordinating side chains are shown as sticks). **E**, Tyr32 and Arg35 (in red), which coordinate the glycerol ligand in the ANDV L_1–200_ structure, are conserved in hantaviruses, but not present in IAV. LACV only possesses the Arginine. The catalytic His36 and the stabilizing Asp37 are shown in bold.

Most of the surface of ANDV L_1–200_ is formed by the helix bundle composed of the N- and C-terminal helices αa+b and αe+f, and the three small helices αc, αc’ and αc” (Fig 1A and 1B). These helices differ between the endonucleases not only in their conformation and orientation, but also in their amino acid composition. Fig 2A shows how this leads to a significant alteration in surface charge distribution compared to the other enzymes. The ANDV endonuclease has large positively charged patches surrounding the active site groove, which are not or only to a lesser extent present in LACV and LCMV or even replaced by negatively charged areas. The more positively charged surface may increase the binding affinity of the enzyme to its substrate, i.e. negatively charged mRNA.

### Active site arrangement and ligand binding

A closer look at the active site of the ANDV endonuclease (Fig 2C) in comparison to LACV and IAV demonstrates the structural similarity of the essential catalytic residues. The side chains of the conserved H-PD-D/E-K motif superimpose well (Fig 2B). The manganese bound to the active site also superimposes with one of the two metal ions present in the other structures. Sequence comparisons suggested that the active site of the ANDV endonuclease is more closely related to IAV than LACV [20, 37]. Indeed, Lys124 of ANDV superimposes with Lys134 of IAV, whereas LACV has a threonine at this position and the equivalent lysine (Lys94) is provided by a different part of the protein.

Close to the active site, electron density for a sulfate and a glycerol molecule is visible in the structure (Fig 2D). The glycerol ligand is coordinated by the conserved residues Tyr32 and Arg35 (Fig 2E), both of which have previously been proposed to regulate the endonuclease activity of the L protein [37].

### Structure–function relationships in the ANDV endonuclease

**Fig 3:**
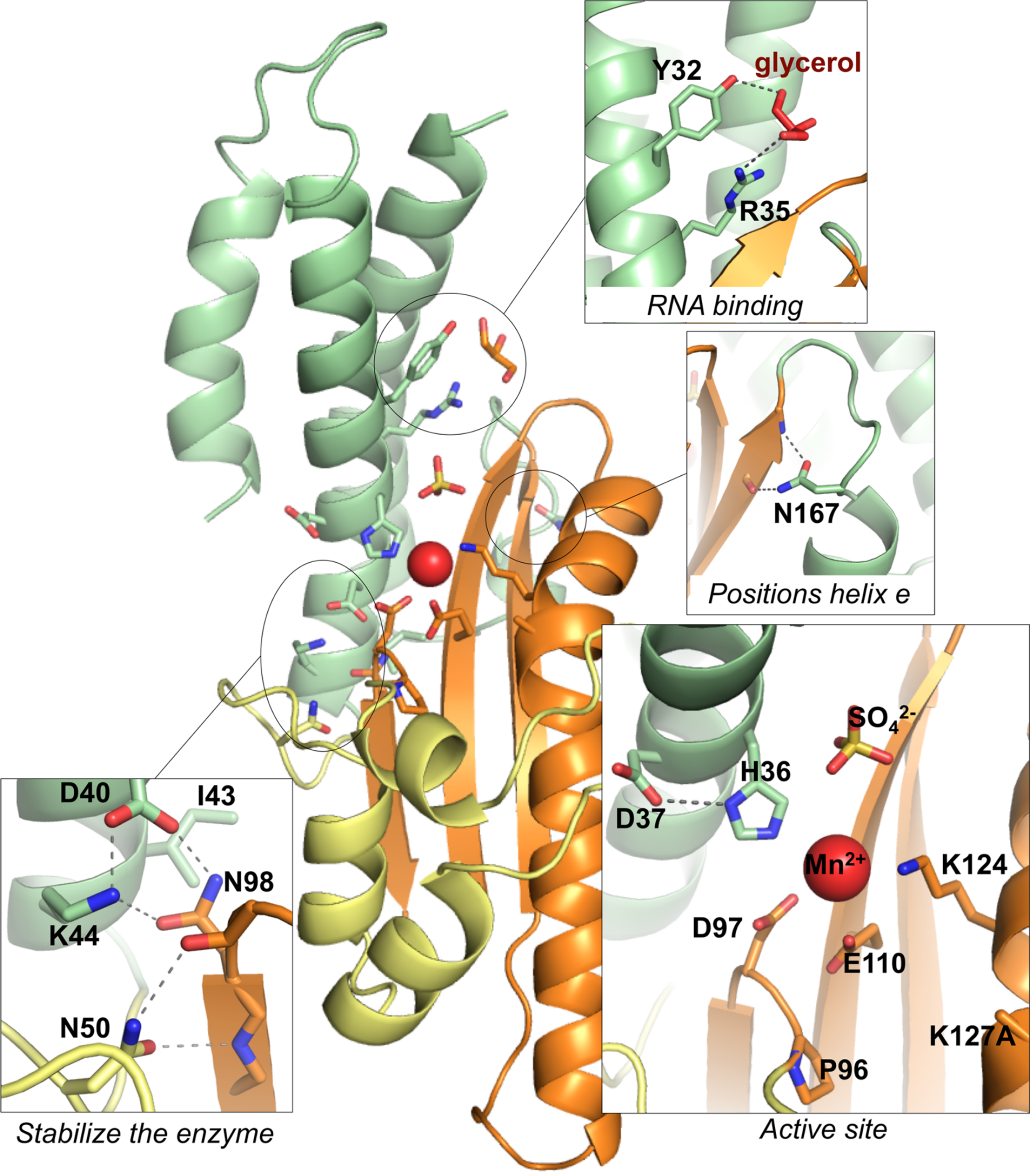
Structure–function relationships in the ANDV endonuclease. Based on the crystal structure of L_1–200_ K127A, a role for the 15 amino acid residues, that have been implicated in the activity of the endonuclease in mammalian cells [37], was inferred. Side chains are shown as sticks and important hydrogen bonds are highlighted with dashed lines. Lys127 is mutated to alanine and therefore not completely represented in the structure. The manganese ion is shown as red sphere.

With the crystal structure of the ANDV cap-snatching endonuclease at hand, we may infer a more precise role for the 15 amino acid residues that have been implicated in the activity of the endonuclease in mammalian cells [37] (Fig 3). The residues can be allocated to five different groups according to their presumed role: (1) catalytic residues (His36, Asp97, and Glu110), (2) active site residues not essential for activity (Asp37, Pro96, Lys124, and Lys127), (3) residues involved in RNA binding in the vicinity of the active site (Tyr32 and Arg35), (4) residues forming stabilizing contacts close to the active site (Asp40, Ile43, Lys44, Asn50, and Asn98), and (5) residues stabilizing helix αe (Asn167). The hypothetical role also considers biochemical and thermal stability data (Table 1 and sections below).

His36, Asp97, and Glu110 (group 1) are the central catalytic residues coordinating the manganese ion (Figs 2C and 3). Asp37 and Pro96 (group 2) are relevant for positioning the catalytic residues His36 and Asp97, respectively. Lys124 and Lys127 (group 2), which emerge from helix αd, may position the RNA substrate in the active site. Tyr32 and Arg35 (group 3) are located in helix αb and bind a glycerol solvent molecule in the structure. Their position at the entrance of the catalytic site and capacity to bind hydroxyl groups let us to speculate that these residues may also be involved in binding the negatively charged RNA substrate. Asp40, Lys44, Asn50, and Asn98 (group 4) form hydrogen bonds with each other and main chain atoms to stabilize and position the loop that contains the catalytic Asp97. Ile43 forms hydrophobic interactions between the end of helix αb and several hydrophobic side chains both from the β-sheet and from helix αe. Group 5 contains only Asn167. It is located in the small helix αe, which is not present in the LACV endonuclease (Fig 1D) and stabilizes this helix via hydrogen bonds with main chain atoms of strand βc.

### Manganese binding and enzymatic activity of the ANDV endonuclease

**Fig 4:**
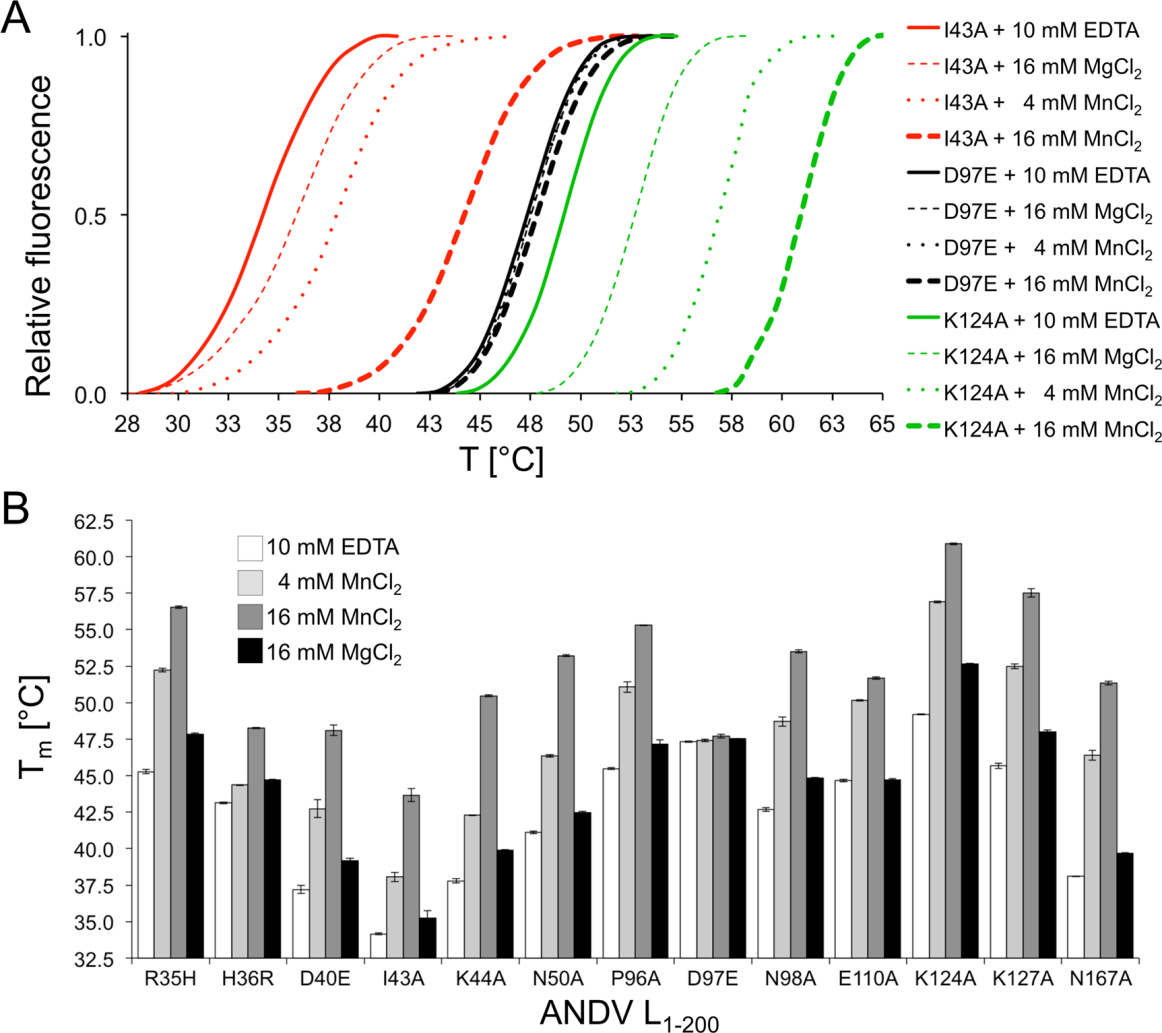
Thermal stability of ANDV L_1–200_ protein mutants. **A**, Representative melting curves for three mutant enzymes. The thermofluor assay was performed in absence of divalent ions (plus 10 mM EDTA), in the presence of manganese (4 mM MnCl_2_ or 16 mM MnCl_2_), and in the presence of magnesium (16 mM MgCl_2_). **B**, Melting temperatures (T_m_) are shown for all mutant enzymes that could be expressed. Protein stability was analyzed in the absence and presence of manganese or magnesium. The data represent mean and standard deviation of four independent thermofluor assays.

The thermal stability of the purified proteins without and with manganese was measured by thermofluor assay [38] in analogy to previously described experiments with the IAV and LACV cap-snatching endonucleases [16, 20]. Melting temperature (T_m_) as a measure of stability varies from 34.2°C for the least stable L_1–200_ I43A mutant to 49.2°C for the most stable L_1–200_ K124A mutant (Fig 4A and 4B). For most mutants, the T_m_ increased by approximately 7°C in the presence of 4 mM manganese, presumably due to binding of the metal in the catalytic site. Consistent with this explanation, mutation of catalytic residues directly involved in metal coordination (His36, Asp97, and Glu110) abolishes this stabilizing effect. Interestingly, increasing the manganese concentration to 16 mM led to a substantial additional T_m_ increase of up to 8°C for some mutants, suggesting binding of another metal ion by the active site (Fig 4A and 4B). In contrast to manganese, magnesium ions showed a much weaker effect (Fig 4A and 4B).

**Fig 5:**
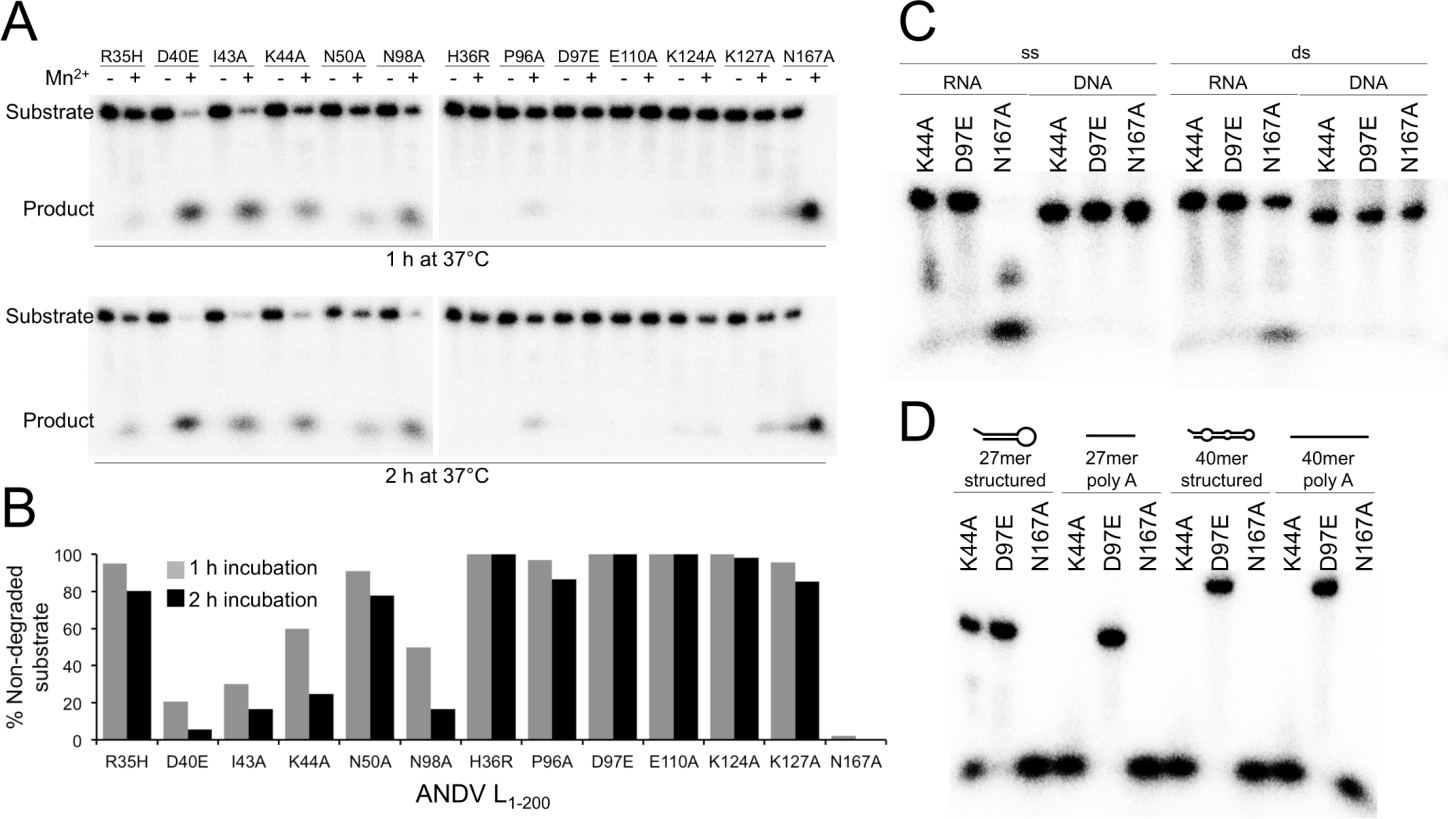
Nuclease activity of ANDV L_1–200_ protein mutants. **A**, Enzyme was incubated with a radioactively labeled 27mer ssRNA in the presence and absence of 2 mM MnCl_2_. Substrate and reaction products were separated on a denaturing polyacrylamide gel. **B**, Signals for residual uncleaved substrate as shown in panel A were quantified using a phosphorimager. **C**, Substrate preference of the enzyme. ANDV L_1–200_ N167A, K44A, and D97E (as a negative control) were incubated with various substrates of identical length and sequence but different structure: single stranded (ss) and double stranded (ds) RNA and DNA. **D**, Enzyme activity with structured ssRNA compared to unstructured (polyA) ssRNA. Both substrates were tested as 27mer and 40mer.

Enzymatic activity was measured in a ribonuclease assay. A 5’-radioactively labeled 27mer single stranded (ss) RNA molecule was used as substrate, and the level of activity was measured by substrate degradation. None of the mutants was active in the absence of divalent ions. Addition of manganese rescued activity for some mutants (Fig 5A), while magnesium had no enhancing effect at all. Therefore, all further assays were performed in the presence of manganese. As expected, active site mutants L_1–200_ H36R, D97E, and E110A were inactive despite addition of manganese (Fig 5A and 5B). Mutation of Arg35, Lys124, and Lys127, still allowed for very low residual activity. Mutation of Asn50 and Pro96 stabilizing the active site loop also strongly reduced endonuclease activity. Mutations affecting stability of the tertiary structure (D40A, I43A, K44A, N98A, and N167A) allowed for intermediate to high activity. We could not detect an intermediate cleavage product of specific length (S4 Fig). The most active mutant was L_1–200_ N167A, which was therefore chosen for further biochemical experimentation. Thermal stability and enzymatic activity data are summarized in Table 1.

### Substrate preference of ANDV endonuclease

To investigate substrate specificity of the ANDV endonuclease, we used three mutants with a range of enzymatic activity: L_1–200_ N167A with high activity, L_1–200_ K44A with intermediate activity, and L_1–200_ D97E as a negative control. First, four different substrates were tested: a 27mer ssRNA that can form secondary structures (predicted hairpin T_m_ = 77°C), the same sequence as double stranded (ds) RNA, and the corresponding ssDNA and dsDNA molecules (Fig 5C). ANDV L_1–200_ N167A and K44A were most active on ssRNA, though they can also less efficiently cleave dsRNA. None of the mutants was active on ssDNA or dsDNA, indicating that the enzyme is a ribonuclease. Furthermore, we compared the ribonuclease activity on ssRNA with different sequence, structure, and length (Fig 5D). Comparison of the 27mer RNA with hairpin-like structure vs. a 27mer unstructured poly(A) RNA reveals preference for the unstructured substrate. In addition, longer RNAs are degraded faster than shorter RNAs. The negative control (L_1–200_ D97E) did not show background degradation, demonstrating that our purification procedure removes potentially contaminating bacterial nucleases.

### Inhibition of ribonuclease activity

**Fig 6:**
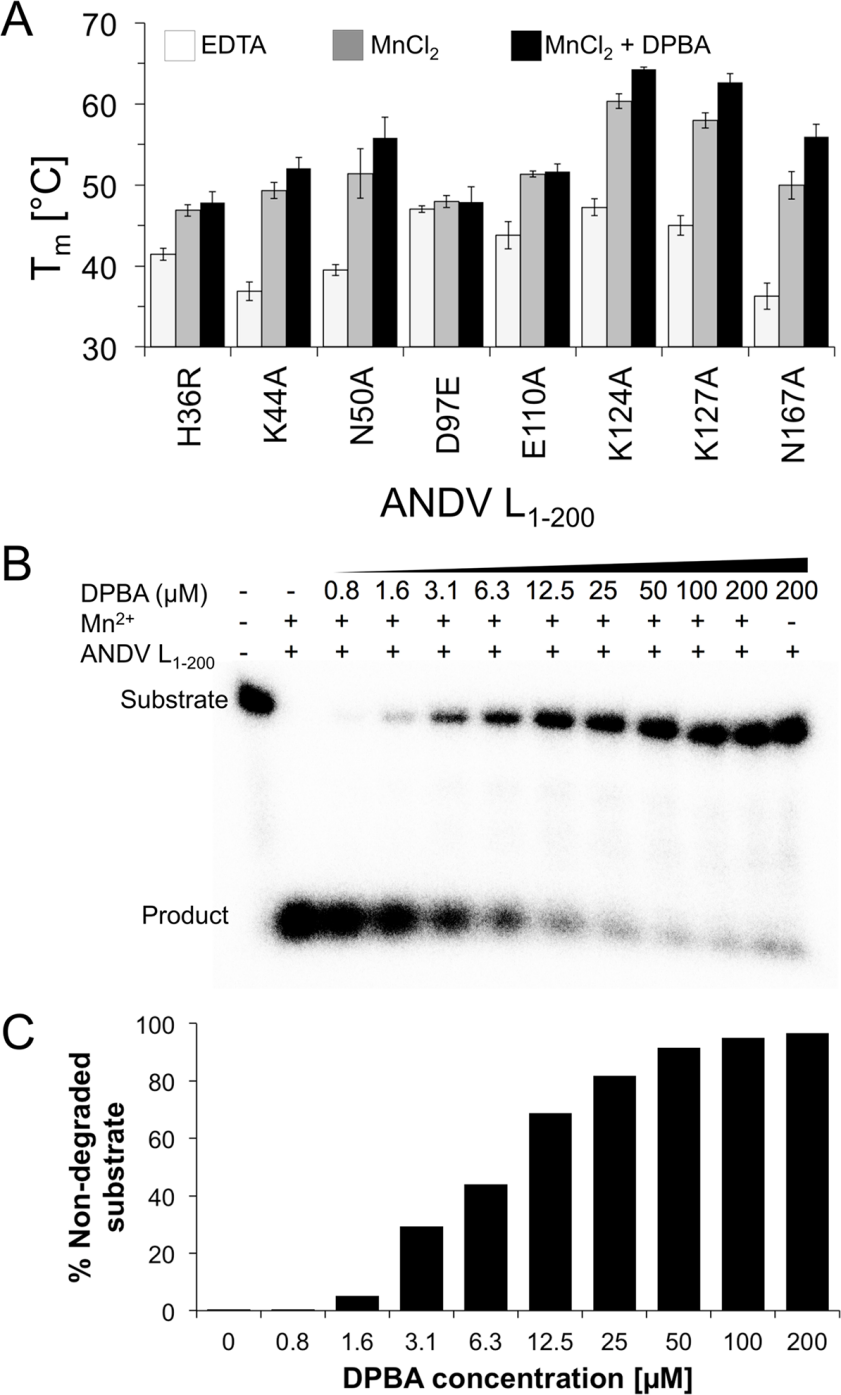
Stabilization and inhibition of ANDV endonuclease by a known endonuclease inhibitor. **A**, Thermal stability of ANDV L_1–200_ proteins in the presence of 16 mM MnCl_2_ and 100 μM 2,4-dioxo-4-phenylbutanoic acid (DPBA) measured in thermofluor assay (T_m_, melting temperature). **B**, RNA nuclease assay with ANDV L_1–200_ N167A in the presence of 2 mM MnCl_2_ and increasing concentrations of DPBA. The inhibitor was incubated with the enzyme for 15 min prior to the addition of the 27mer ssRNA substrate. The assay was performed for 1 h at 37°C. Substrate and reaction products were separated on a denaturing polyacrylamide gel. **C**, Signals for residual uncleaved substrate as shown in panel A were quantified using a phosphorimager.

The metal-dependent cap-snatching endonuclease is an attractive target for antiviral drugs against segmented negative strand RNA viruses and promising compounds have been described for influenza [24–35]. To proof suitability of the ANDV endonuclease expressed in this study for screening of potential inhibitors, we first measured the effect of the classical influenza virus endonuclease inhibitor 2,4-dioxo-4-phenylbutanoic acid (DPBA) [33] on thermal stability of ANDV L_1–200_ mutants H36R, K44A, N50A, D97E, E110A, K125A, K127A, and N167A in the presence of 16 mM MnCl_2_. All mutants that showed a stabilization of > 8°C in the presence of manganese, also showed a further increase in the melting temperature upon addition of DPBA (Fig 6A), indicating that DPBA binds to and stabilizes the manganese–active site complex. In agreement with this conclusion, mutation of residues His36, Asp97 and Glu110, which are involved in coordination of at least one manganese ion, abolishes the stabilizing effect of DPBA.

To prove that DPBA not just binds to the active site of ANDV L_1–200_, but also inhibits the ribonuclease activity, we incubated the most active mutant ANDV L_1–200_ N167A with Mn^2+^ and DPBA for 15 min prior to the addition of the RNA substrate. Fig 6B and 6C show a clear dose-dependent inhibition of the enzymatic activity comparable to IAV and LACV [16, 20].

## Discussion

This study provides structural and biochemical evidence for the existence of an endonuclease in the N terminus of ANDV L protein. In agreement with previous studies on expression of L protein in mammalian cells [36, 37], we have not been able to express the wild-type endonuclease domain in bacterial cells. Therefore, we took advantage of a range of mutations to facilitate expression of the domain. By using this approach, we expressed large amounts of various mutants and eventually solved the crystal structure of the ANDV L_1–200_ K127A mutant. The first structure of a hantavirus cap-snatching endonuclease reveals similarities to the related enzymes from LACV, LCMV, and IAV [16, 18, 20–23]. Besides the active site residues the sequence is hardly conserved. However there are obvious common structural features present: all cap-snatching endonucleases consist of two lobes with the conserved active site buried in a cavity in between. However, compared to the other enzymes, ANDV has positively charged patches surrounding the active site. The positively charged surface may increase the affinity of the enzyme for negatively charged RNA substrates. Whether or not this feature plays a role in the apparently strong endonuclease activity upon recombinant expression remains to be determined.

The structure in conjunction with the thermal stability and enzymatic activity data of the attenuated mutants allow us to speculate on the contribution of individual residues for enzymatic activity, substrate binding, and overall stability of the tertiary structure (Table 1). The data demonstrate that residues His36, Asp97, and Glu110 coordinate the manganese ion and are essential for the catalytic process. All three residues act in a cooperative manner, as removal of one residue is sufficient to prevent binding of both metal ions and inactivate the enzyme. The two lysine residues at positions 124 and 127 in the active site may stabilize the attacking hydroxide nucleophile during catalysis and/or bind to the RNA substrate, as speculated previously for IAV and LACV [16, 20]. Mutation of both lysines does not affect the stabilizing effect of DPBA, suggesting they play no essential role in metal binding. The two residues Tyr32 and Arg35, which coordinate a glycerol ligand in the crystal structure, may also be involved in substrate binding. Arg35 seems to be of particular relevance, as it is conserved in all bunyavirus L proteins and the L_1–200_ R35H mutant is largely defective, although it clearly binds manganese in the thermal stability experiments. Tyr32 seems to be less important, as the L_1–200_ Y32V mutant behaves like the wild-type protein and is toxic to *E*. *coli* cells.

Interestingly, several attenuating mutations (D40E, I43A, K44A, N50A, N98A, and N167A) seem to slightly destabilize the protein, which might affect the enzymatic activity of the enzyme. The corresponding wild-type residues are involved in hydrogen bonds with other side or main chain atoms or in hydrophobic interactions with neighboring secondary structure elements. Consistent with the structural data, most of these mutants are characterized by low temperature stability and high residual enzymatic activity. Residue Asn167 is exceptional as it is located distant to the active site. Via hydrogen bonds with β-sheet βc it positions a small helix (αe) that is not present in the otherwise similar LACV protein. Consistent with the peripheral location of Asn167, the corresponding mutant is the least attenuated. Its wild-type like features include the highest enzymatic activity and a reduced growth rate of the expressing *E*. *coli* cells. Given the strong activity of an attenuated version of the enzyme on various RNA templates, it is plausible that the wild-type enzyme activity is too toxic for high-level expression in pro- and eukaryotic cells. Hantaviruses must have an intricate mechanism to control the activity of the endonuclease in infected cells or to keep the concentration of the L protein at a sub-toxic level.

The biochemical data also revealed a clear co-factor and substrate specificity of the ANDV endonuclease. Consistent with its presumed function as a cap-snatching enzyme, it preferred ssRNA over dsRNA substrates and was inactive on ssDNA or dsDNA. RNA length and secondary structure seem to influence substrate binding. Dependence of enzymatic activity from manganese rather than magnesium has also been observed for the endonucleases of the other segmented negative strand viruses [16, 20, 21]. However, in contrast to LACV and IAV with two metal ions in the active site [16, 20], the ANDV crystal structure contains only a single manganese ion (equivalent to Mn1) [21], while the LCMV structure does not contain any ions in the active site. The reason for this discrepancy may be explained by our crystallization conditions and thermal stability data. At 2 mM manganese, the active site in the crystals contained a single ion. However, we observed an additional increase in thermal stability when increasing the manganese concentration from 4 mM to 16 mM, suggesting the binding of an additional ion (equivalent to Mn2) at higher manganese concentration. This is in agreement with findings by Reguera et al. 2016 [39] for related endonucleases showing a high binding affinity for the first manganese ion (Mn1), but low affinity for the second manganese ion (Mn2). It is reasonable to propose that ANDV and other hantaviruses share the same two-metal dependent catalytic mechanism with LACV and IAV [16].

This study also has implications for development of antiviral drugs to treat hantavirus infections. The influenza virus endonuclease has been used as a target in antiviral drug discovery programs and several specific inhibitors have been found [24–35]. Likewise, the ANDV endonuclease is an attractive target for antiviral drug development. The experiments with the known endonuclease inhibitor DPBA [33] provide proof-of-concept that the ribonuclease activity of the enzyme is amenable to compound screening. The highly active ANDV L_1–200_ N167A mutant is the most promising enzyme candidate for this purpose.

## Materials and Methods

### Cloning, expression, and purification of the N-terminal domain of ANDV L protein

The cDNA of fragments encoding residues 1–163, 1–179, 1–191, 1–194, 1–197, 1–200, 1–211, 1–214 and 1–228 from the L protein of ANDV (strain Chile 9717869, GenBank accession no. AF291704) were amplified by PCR using the pCITE-ANDV-L plasmid [37] as a template. Virus sequences were cloned into pOPIN-F (N-terminal His-tag–3C protease cleavage site) and pOPIN-M (N-terminal His-tag–maltose binding protein–3C protease cleavage site) [41]. L protein mutants (Y32V, R35H, H36R, D37A, D40E, I43A, K44A, N50A, P96A, D97E, N98A, E110A, K124A, K127A and N167A) were generated via a classical two-step PCR mutagenesis approach as described previously [42] and the resulting fragments cloned into pOPIN-F. For cloning, *E*. *coli* strain DH5α was used. The sequence of the inserts was confirmed by sequencing.

Proteins were expressed in *E*. *coli* strain BL21 Gold (DE3) (Novagen) at 17°C overnight using LB medium and 0.5 mM isopropyl-β-D-thiogalactopyranosid for induction. Cells were pelleted, resuspended in lysis buffer containing 50 mM Tris-HCl pH 7.3, 300 mM NaCl, 10 mM imidazole, 10% glycerol, 10 mM MnCl_2_, and 1 mM phenylmethylsulfonyl fluorid and disrupted by sonication. The soluble material was loaded onto a Ni-NTA affinity column, washed with 10 column volumes of lysis buffer containing 50 mM imidazole, and eluted with 5 volumes of lysis buffer containing 500 mM imidazole. The His-tag of eluted protein was cleaved off using glutathione S-transferase-tagged 3C protease at 4°C overnight during dialysis against lysis buffer. The untagged proteins were further purified by size exclusion chromatography using a Superdex 200 column in 50 mM sodium citrate pH 5.5, 1 M NaCl, 5% glycerol. Purified proteins were concentrated using centrifugal devices, flash frozen in liquid nitrogen, and stored in aliquots at –20°C.

### Crystallization and structure determination

ANDV L_1–200_ K127A (10 mg/ml) was crystallized in the presence of 2 mM MnCl_2_, 100 mM sodium acetate pH 5.0, 27.5% polyethylene glycol 3350, and 500 mM (NH_4_)_2_SO_4_ by sitting drop vapor diffusion. Crystals were flash frozen in liquid nitrogen with 8% butanediol as cryo protectant and diffraction data were collected to 2.4 Å at beamline ID23-1 at the European Synchrotron Radiation Facility, Grenoble, with a wavelength of 0.98 Å and 1.77 Å for respectively native and anomalous data. Datasets were processed with iMosflm [43]. The crystal structure was solved in space group P42_1_2 by molecular replacement using residues 32–162 from the endonuclease from Hantaan virus [39] and PHASER [44], and refined by iterative cycles of manual model building in Coot [45] and computational optimization with PHENIX [46]. TLS refinement was performed during the final stages of refinement [47] with the entire molecule as rigid group. Data collection and refinement statistics are shown in the crystallographic table in the supplemental information. Structural data were visualized with Pymol and Chimera [48]. Electrostatic surfaces were calculated using PDB2PQR and APBS [49, 50]. The coordinates have been deposited to the PDB (5HSB.pdb).

### Small angle X-ray scattering

Small angle X-ray scattering (SAXS) data for 1, 2, and 5 mg/ml of ANDV L_1–200_ K127A in 2 mM MnCl_2_, 50 mM Tris-HCl pH 7.3, 250 mM NaCl, 5% glycerol were collected at the SAXS beamline P12 at the PETRA III storage ring of the Deutsches Elektronen-Synchrotron, Hamburg [51]. Using a PILATUS 2M pixel detector at 3.1 m sample distance and 10 keV energy (λ = 1.24 Å), a momentum transfer range of 0.01 Å^–1^ < s < 0.45 Å^–1^ was covered (s = 4π sinθ/λ, where 2θ is the scattering angle). Data were analyzed using the ATSAS 2.6 package [52]. The forward scattering I(0) and the radius of gyration R_g_ were extracted from the Guinier approximation calculated with the AutoRG function within PRIMUS [53]. GNOM [54] provided the pair distribution function P(r) of the particle and the maximum size D_max_. *Ab initio* reconstructions were generated with the program DAMMIF [55]. Ten independent DAMMIF runs were superimposed by SUPCOMB [56] and averaged using the program DAMAVER [55].

### Nuclease assay

Nuclease activity was measured by incubating 1 μM ANDV L_1–200_ protein with 0.1 μM ^32^P-labeled single stranded (ss) or double stranded (ds) 27mer RNA or DNA substrate (5’-GA^U^/_T_GA^U^/_T_GC^U^/_T_A^U^/_T_CACCGCGC^U^/_T_CG^U^/_T_CG^U^/_T_C-3’) or 40mer ssRNA substrate (5´-GAUGAUGCUAUCACCGCGCUCGUCGUCGAUGAUGCUAUCA-3’) in 50 mM Tris-HCl pH 7.3, 250 mM NaCl, 5% glycerol, 0.25 U/μl RNasin (Promega) in the absence or presence of 2 mM MnCl_2_ at 37°C for 1 or 2 h. RNA was annealed by heating to 98°C for 2 minutes, followed by a slow cooling over 2 hours to room temperature. A 27 or 40mer poly(A) ssRNA was used as unstructured substrate. In selected experiments, 2,4-dioxo-4-phenylbutanoic acid (DPBA) was added to the assay. The reaction was stopped by adding 2 × loading buffer (95% formamide, 18 mM ethylenediaminetetraacetic acid (EDTA), 0.025% sodium dodecyl sulfate, xylene cyanol, and bromophenol blue) and heating the samples to 98°C for 5 min. The reaction products were separated by 8 M urea, 20% polyacrylamide, Tris-borate-EDTA gel electrophoresis and visualized by phosphor screen autoradiography using a Typhoon scanner (GE Healthcare). Intensity of the signals was quantified by ImageJ software [57].

### Thermal stability assay

The stability of ANDV L_1–200_ protein was measured by thermofluor assay at a protein concentration of 10 μM in 100 mM Tris pH 7.0, 250 mM NaCl, 5% glycerol complemented with either 10 mM EDTA, 4 mM MnCl_2_, 16 mM MnCl_2_, 16 mM MgCl_2_ or 16 mM MnCl_2_ plus 100 μM DPBA as described [38].

## Supporting Information

S1 Fig Purified ANDV L_1–200_ mutants used in the study. Following purification, 1 μg of protein was separated by sodium dodecyl sulfate polyacrylamide gel electrophoresis and stained with Coomassie. The His-tag of ANDV L_1–200_ K127A had been cleaved off for crystallization experiments. All other proteins feature an N-terminal His-tag.
(PDF)

S2 Fig Buffer optimization for ANDV L_1–200_ K127A using thermofluor assay. The stability of the purified protein was tested in thermofluor assay under various conditions. The protein is most stable at lower pH and higher salt concentrations.
(PDF)

S3 Fig Small-angle X-ray scattering (SAXS) data of ANDV L_1-200_ K127A. Experimental scattering curves (black dots) are compared with theoretical scattering curves for the ANDV crystal structure (black line), the LACV crystal structure (blue line), the LCMV crystal structure (green line) and the IAV crystal structure (orange line). χ^2^ –values of fits are given for each structure, curves were calculated with CRYSOL [58].
(PDF)

S4 Fig Time dependency of endonucleolytic cleavage. Degradation of unstructured polyA RNA by ANDV L_1-200_ N167A shows no accumulation of larger intermediate RNA products with specific lengths.
(PDF)

S1 Table Crystallographic data collection and refinement statistics. (PDF)

## References

[1] Macneil A, Nichol ST, Spiropoulou CF. Hantavirus pulmonary syndrome. Virus Res. 2011;162(1–2):138–47. 10.1016/j.virusres.2011.09.017 21945215

[2] Jonsson CB, Figueiredo LT, Vapalahti O. A global perspective on hantavirus ecology, epidemiology, and disease. Clin Microbiol Rev. 2010;23(2):412–41. 10.1128/CMR.00062-09 20375360 PMC2863364

[3] Manigold T, Vial P. Human hantavirus infections: epidemiology, clinical features, pathogenesis and immunology. Swiss Med Wkly. 2014;144:w13937 10.4414/smw.2014.13937 24652684

[4] Schountz T, Prescott J. Hantavirus immunology of rodent reservoirs: current status and future directions. Viruses. 2014;6(3):1317–35. 10.3390/v6031317 24638205 PMC3970152

[5] Medina RA, Torres-Perez F, Galeno H, Navarrete M, Vial PA, Palma RE, et al Ecology, genetic diversity, and phylogeographic structure of andes virus in humans and rodents in Chile. J Virol. 2009;83(6):2446–59. 10.1128/JVI.01057-08 19116256 PMC2648280

[6] Wells RM, Young J, Williams RJ, Armstrong LR, Busico K, Khan AS, et al Hantavirus transmission in the United States. Emerg Infect Dis. 1997;3(3):361–5. 9284382 10.3201/eid0303.970314 PMC2627629

[7] Martinez VP, Bellomo C, San Juan J, Pinna D, Forlenza R, Elder M, et al Person-to-person transmission of Andes virus. Emerg Infect Dis. 2005;11(12):1848–53. 16485469 10.3201/eid1112.050501 PMC3367635

[8] Hepojoki J, Strandin T, Lankinen H, Vaheri A. Hantavirus structure—molecular interactions behind the scene. J Gen Virol. 2012;93(Pt 8):1631–44. 10.1099/vir.0.042218-0 22622328

[9] Kukkonen SK, Vaheri A, Plyusnin A. L protein, the RNA-dependent RNA polymerase of hantaviruses. Arch Virol. 2005;150(3):533–56. 15503219 10.1007/s00705-004-0414-8

[10] Cimica V, Dalrymple NA, Roth E, Nasonov A, Mackow ER. An innate immunity-regulating virulence determinant is uniquely encoded by the Andes virus nucleocapsid protein. MBio. 2014;5(1). 10.1128/mBio.01088-13 PMC3944819 24549848

[11] Jaaskelainen KM, Kaukinen P, Minskaya ES, Plyusnina A, Vapalahti O, Elliott RM, et al Tula and Puumala hantavirus NSs ORFs are functional and the products inhibit activation of the interferon-beta promoter. J Med Virol. 2007;79(10):1527–36. 17705180 10.1002/jmv.20948

[12] Cheng E, Mir MA. Signatures of host mRNA 5' terminus for efficient hantavirus cap snatching. J Virol. 2012;86(18):10173–85. 10.1128/JVI.05560-11 22787213 PMC3446632

[13] Garcin D, Lezzi M, Dobbs M, Elliott RM, Schmaljohn C, Kang CY, et al The 5' ends of Hantaan virus (Bunyaviridae) RNAs suggest a prime-and-realign mechanism for the initiation of RNA synthesis. J Virol. 1995;69(9):5754–62. 7637020 10.1128/jvi.69.9.5754-5762.1995 PMC189436

[14] Decroly E, Ferron F, Lescar J, Canard B. Conventional and unconventional mechanisms for capping viral mRNA. Nat Rev Microbiol. 2012;10(1):51–65. 10.1038/nrmicro2675 PMC7097100 22138959

[15] Plotch SJ, Bouloy M, Ulmanen I, Krug RM. A unique cap(m7GpppXm)-dependent influenza virion endonuclease cleaves capped RNAs to generate the primers that initiate viral RNA transcription. Cell. 1981;23(3):847–58. 6261960 10.1016/0092-8674(81)90449-9

[16] Dias A, Bouvier D, Crepin T, McCarthy AA, Hart DJ, Baudin F, et al The cap-snatching endonuclease of influenza virus polymerase resides in the PA subunit. Nature. 2009;458(7240):914–8. 10.1038/nature07745 19194459

[17] Pflug A, Guilligay D, Reich S, Cusack S. Structure of influenza A polymerase bound to the viral RNA promoter. Nature. 2014;516(7531):355–60. 10.1038/nature14008 25409142

[18] Guilligay D, Tarendeau F, Resa-Infante P, Coloma R, Crepin T, Sehr P, et al The structural basis for cap binding by influenza virus polymerase subunit PB2. Nat Struct Mol Biol. 2008;15(5):500–6. 10.1038/nsmb.1421 18454157

[19] Reich S, Guilligay D, Pflug A, Malet H, Berger I, Crepin T, et al Structural insight into cap-snatching and RNA synthesis by influenza polymerase. Nature. 2014;516(7531):361–6. 10.1038/nature14009 25409151

[20] Reguera J, Weber F, Cusack S. Bunyaviridae RNA polymerases (L-protein) have an N-terminal, influenza-like endonuclease domain, essential for viral cap-dependent transcription. PLoS Pathog. 2010;6(9):e1001101 10.1371/journal.ppat.1001101 20862319 PMC2940753

[21] Morin B, Coutard B, Lelke M, Ferron F, Kerber R, Jamal S, et al The N-terminal domain of the arenavirus L protein is an RNA endonuclease essential in mRNA transcription. PLoS Pathog. 2010;6(9):e1001038 10.1371/journal.ppat.1001038 20862324 PMC2940758

[22] Wallat GD, Huang Q, Wang W, Dong H, Ly H, Liang Y, et al High-resolution structure of the N-terminal endonuclease domain of the Lassa virus L polymerase in complex with magnesium ions. PLoS One. 2014;9(2):e87577 10.1371/journal.pone.0087577 24516554 PMC3917842

[23] Gerlach P, Malet H, Cusack S, Reguera J. Structural Insights into Bunyavirus Replication and Its Regulation by the vRNA Promoter. Cell. 2015;161(6):1267–79. 10.1016/j.cell.2015.05.006 26004069 PMC4459711

[24] Fudo S, Yamamoto N, Nukaga M, Odagiri T, Tashiro M, Neya S, et al Structural and computational study on inhibitory compounds for endonuclease activity of influenza virus polymerase. Bioorg Med Chem. 2015;23(17):5466–75. 10.1016/j.bmc.2015.07.046 26252962

[25] Hsu JT, Yeh JY, Lin TJ, Li ML, Wu MS, Hsieh CF, et al Identification of BPR3P0128 as an inhibitor of cap-snatching activities of influenza virus. Antimicrob Agents Chemother. 2012;56(2):647–57. 10.1128/AAC.00125-11 21930871 PMC3264257

[26] Kowalinski E, Zubieta C, Wolkerstorfer A, Szolar OH, Ruigrok RW, Cusack S. Structural analysis of specific metal chelating inhibitor binding to the endonuclease domain of influenza pH1N1 (2009) polymerase. PLoS Pathog. 2012;8(8):e1002831 10.1371/journal.ppat.1002831 22876177 PMC3410856

[27] Kuzuhara T, Iwai Y, Takahashi H, Hatakeyama D, Echigo N. Green tea catechins inhibit the endonuclease activity of influenza A virus RNA polymerase. PLoS Curr. 2009;1:RRN1052 20025206 10.1371/currents.RRN1052 PMC2762814

[28] Nakazawa M, Kadowaki SE, Watanabe I, Kadowaki Y, Takei M, Fukuda H. PA subunit of RNA polymerase as a promising target for anti-influenza virus agents. Antiviral Res. 2008;78(3):194–201. 10.1016/j.antiviral.2007.12.010 18258312

[29] Pala N, Stevaert A, Dallocchio R, Dessi A, Rogolino D, Carcelli M, et al Virtual Screening and Biological Validation of Novel Influenza Virus PA Endonuclease Inhibitors. ACS Med Chem Lett. 2015;6(8):866–71. 10.1021/acsmedchemlett.5b00109 26288686 PMC4538433

[30] Parkes KE, Ermert P, Fassler J, Ives J, Martin JA, Merrett JH, et al Use of a pharmacophore model to discover a new class of influenza endonuclease inhibitors. J Med Chem. 2003;46(7):1153–64. 12646026 10.1021/jm020334u

[31] Sagong HY, Bauman JD, Patel D, Das K, Arnold E, LaVoie EJ. Phenyl substituted 4-hydroxypyridazin-3(2H)-ones and 5-hydroxypyrimidin-4(3H)-ones: inhibitors of influenza A endonuclease. J Med Chem. 2014;57(19):8086–98. 10.1021/jm500958x 25225968 PMC4191602

[32] Shibagaki Y, Ikuta N, Iguchi S, Takaki K, Watanabe S, Kaihotsu M, et al An efficient screening system for influenza virus cap-dependent endonuclease inhibitors. J Virol Methods. 2014;202:8–14. 10.1016/j.jviromet.2014.02.005 24613941

[33] Tomassini J, Selnick H, Davies ME, Armstrong ME, Baldwin J, Bourgeois M, et al Inhibition of cap (m7GpppXm)-dependent endonuclease of influenza virus by 4-substituted 2,4-dioxobutanoic acid compounds. Antimicrob Agents Chemother. 1994;38(12):2827–37. 7695269 10.1128/aac.38.12.2827 PMC188292

[34] Tomassini JE, Davies ME, Hastings JC, Lingham R, Mojena M, Raghoobar SL, et al A novel antiviral agent which inhibits the endonuclease of influenza viruses. Antimicrob Agents Chemother. 1996;40(5):1189–93. 8723464 10.1128/aac.40.5.1189 PMC163289

[35] DuBois RM, Slavish PJ, Baughman BM, Yun MK, Bao J, Webby RJ, et al Structural and biochemical basis for development of influenza virus inhibitors targeting the PA endonuclease. PLoS Pathog. 2012;8(8):e1002830 10.1371/journal.ppat.1002830 22876176 PMC3410894

[36] Cheng E, Wang Z, Mir MA. Interaction between hantavirus nucleocapsid protein (N) and RNA-dependent RNA polymerase (RdRp) mutants reveals the requirement of an N-RdRp interaction for viral RNA synthesis. J Virol. 2014;88(15):8706–12. 10.1128/JVI.00405-14 24850733 PMC4135928

[37] Heinemann P, Schmidt-Chanasit J, Gunther S. The N terminus of Andes virus L protein suppresses mRNA and protein expression in mammalian cells. J Virol. 2013;87(12):6975–85. 10.1128/JVI.00043-13 23576516 PMC3676134

[38] Ericsson UB, Hallberg BM, Detitta GT, Dekker N, Nordlund P. Thermofluor-based high-throughput stability optimization of proteins for structural studies. Anal Biochem. 2006;357(2):289–98. 16962548 10.1016/j.ab.2006.07.027

[39] Reguera J, Gerlach P, Rosenthal M, Gaudon S, Coscia F, Guenther S, et al Comparative structural and functional analysis of bunya- and arenavirus cap-snatching endonucleases. co-submission to Plos Pathogens. 2016. 10.1371/journal.ppat.1005636 PMC4909276 27304209

[40] Holm L, Kaariainen S, Wilton C, Plewczynski D. Using Dali for structural comparison of proteins. Curr Protoc Bioinformatics. 2006;Chapter 5:Unit 5 10.1002/0471250953.bi0505s14 18428766

[41] Berrow NS, Alderton D, Sainsbury S, Nettleship J, Assenberg R, Rahman N, et al A versatile ligation-independent cloning method suitable for high-throughput expression screening applications. Nucleic Acids Research. 2007;35(6). 10.1093/nar/gkm047 PMC1874605 17317681

[42] Hass M, Lelke M, Busch C, Becker-Ziaja B, Gunther S. Mutational evidence for a structural model of the Lassa virus RNA polymerase domain and identification of two residues, Gly1394 and Asp1395, that are critical for transcription but not replication of the genome. J Virol. 2008;82(20):10207–17. 10.1128/JVI.00220-08 18667512 PMC2566270

[43] Battye TGG, Kontogiannis L, Johnson O, Powell HR, Leslie AGW. iMOSFLM: a new graphical interface for diffraction-image processing with MOSFLM. Acta Crystallographica Section D-Biological Crystallography. 2011;67:271–81. 10.1107/S0907444910048675 PMC3069742 21460445

[44] McCoy AJ, Grosse-Kunstleve RW, Adams PD, Winn MD, Storoni LC, Read RJ. Phaser crystallographic software. J Appl Crystallogr. 2007;40(Pt 4):658–74. 19461840 10.1107/S0021889807021206 PMC2483472

[45] Emsley P, Lohkamp B, Scott WG, Cowtan K. Features and development of Coot. Acta Crystallographica Section D-Biological Crystallography. 2010;66:486–501. 10.1107/S0907444910007493 PMC2852313 20383002

[46] Adams PD, Afonine PV, Bunkoczi G, Chen VB, Davis IW, Echols N, et al PHENIX: a comprehensive Python-based system for macromolecular structure solution. Acta Crystallogr D Biol Crystallogr. 2010;66(Pt 2):213–21. 10.1107/S0907444909052925 20124702 PMC2815670

[47] Painter J, Merritt EA. Optimal description of a protein structure in terms of multiple groups undergoing TLS motion. Acta Crystallogr D Biol Crystallogr. 2006;62(Pt 4):439–50. 16552146 10.1107/S0907444906005270

[48] Pettersen EF, Goddard TD, Huang CC, Couch GS, Greenblatt DM, Meng EC, et al UCSF Chimera—a visualization system for exploratory research and analysis. J Comput Chem. 2004;25(13):1605–12. 15264254 10.1002/jcc.20084

[49] Baker NA, Sept D, Joseph S, Holst MJ, McCammon JA. Electrostatics of nanosystems: application to microtubules and the ribosome. Proc Natl Acad Sci U S A. 2001;98(18):10037–41. 11517324 10.1073/pnas.181342398 PMC56910

[50] Dolinsky TJ, Czodrowski P, Li H, Nielsen JE, Jensen JH, Klebe G, et al PDB2PQR: expanding and upgrading automated preparation of biomolecular structures for molecular simulations. Nucleic Acids Res. 2007;35(Web Server issue):W522–5. 17488841 10.1093/nar/gkm276 PMC1933214

[51] Blanchet CE, Spilotros A, Schwemmer F, Graewert MA, Kikhney A, Jeffries CM, et al Versatile sample environments and automation for biological solution X-ray scattering experiments at the P12 beamline (PETRA III, DESY). J Appl Crystallogr. 2015;48(Pt 2):431–43. 25844078 10.1107/S160057671500254X PMC4379436

[52] Petoukhov MV, Franke D, Shkumatov AV, Tria G, Kikhney AG, Gajda M, et al New developments in the program package for small-angle scattering data analysis. J Appl Crystallogr. 2012;45(Pt 2):342–50. 25484842 10.1107/S0021889812007662 PMC4233345

[53] Konarev PV, Volkov VV, Sokolova AV, Koch MHJ, Svergun DI. PRIMUS: a Windows PC-based system for small-angle scattering data analysis. Journal of Applied Crystallography. 2003;36:1277–82.

[54] Svergun DI. Determination of the regularization parameter in indirect-transform methods using perceptual criteria. J Appl Crystallogr. 1992;25:495–503.

[55] Volkov VV, Svergun DI. Uniqueness of ab initio shape determination in small-angle scattering. Journal of Applied Crystallography. 2003;36:860–4. 10.1107/S0021889809000338 PMC5023043 27630371

[56] Kozin MB, Svergun DI. Automated matching of high- and low-resolution structural models. Journal of Applied Crystallography. 2001;34:33–41.

[57] Schneider CA, Rasband WS, Eliceiri KW. NIH Image to ImageJ: 25 years of image analysis. Nat Methods. 2012;9(7):671–5. 22930834 10.1038/nmeth.2089 PMC5554542

[58] Svergun D, Barberato C, Koch MHJ. CRYSOL—A program to evaluate x-ray solution scattering of biological macromolecules from atomic coordinates. Journal of Applied Crystallography. 1995;28:768–73.

