# Supplementary figures and images for: Atomic Structure and Biochemical Characterization of an RNA Endonuclease in the N Terminus of Andes Virus L Protein
Source: PLoS Pathog. 2016 Jun 14;12(6):e1005635. doi: 10.1371/journal.ppat.1005635 (PMC4907427; doi:10.1371/journal.ppat.1005635)

S1 Figure

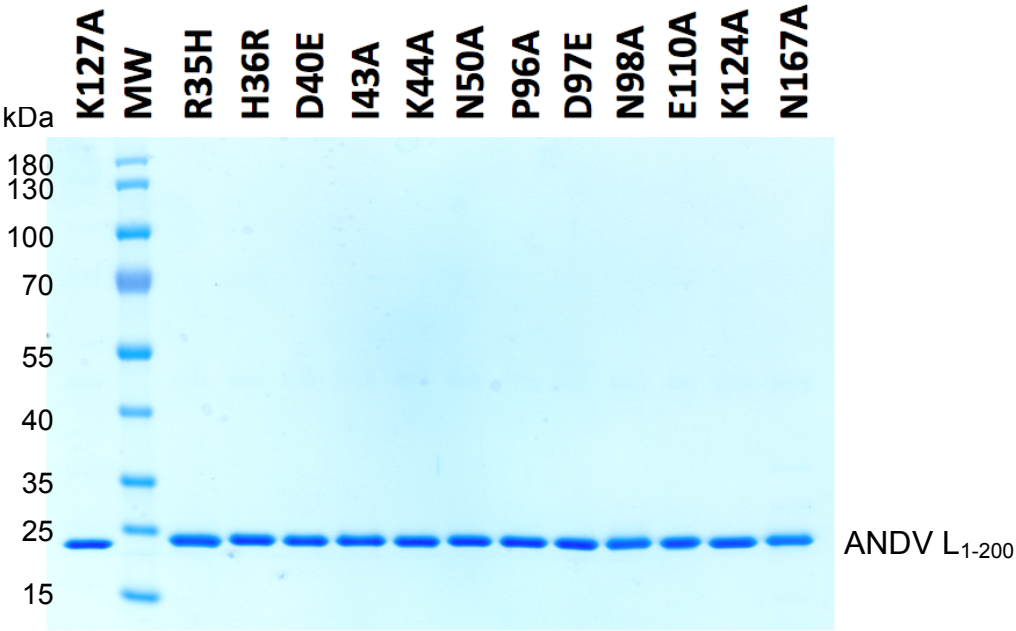

Supplement: S1 Fig — Following purification, 1 μg of protein was separated by sodium dodecyl sulfate polyacrylamide gel electrophoresis and stained with Coomassie. The His-tag of ANDV L1–200 K127A had been cleaved off for crystallization experiments. All other proteins feature an N-terminal His-tag. (PDF) [file ppat.1005635.s001.pdf]

**S2 Figure**

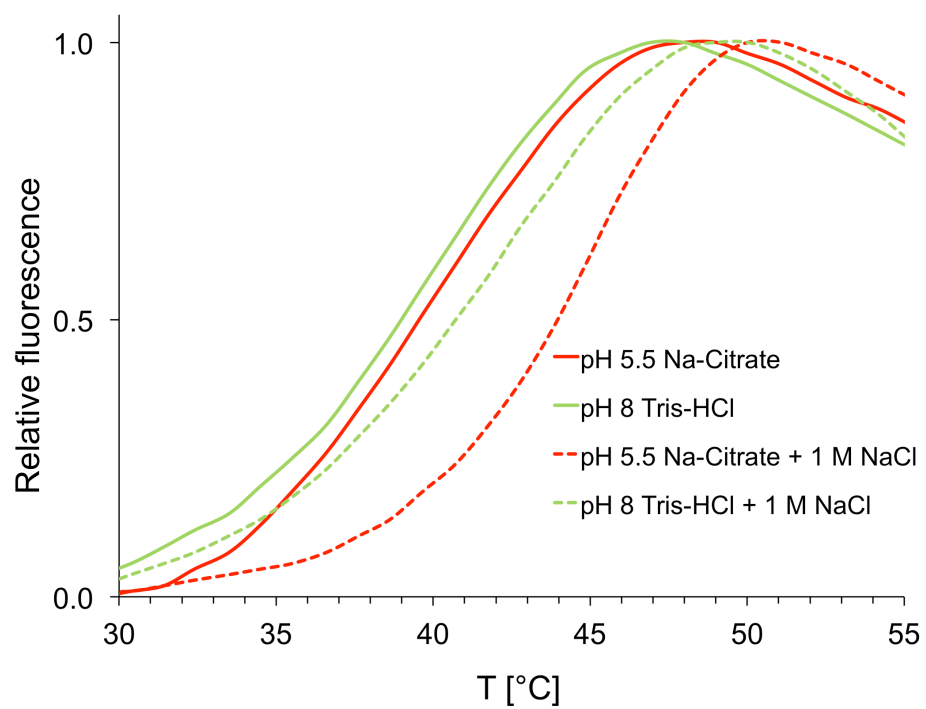

Supplement: S2 Fig — The stability of the purified protein was tested in thermofluor assay under various conditions. The protein is most stable at lower pH and higher salt concentrations. (PDF) [file ppat.1005635.s002.pdf]

S3 Figure

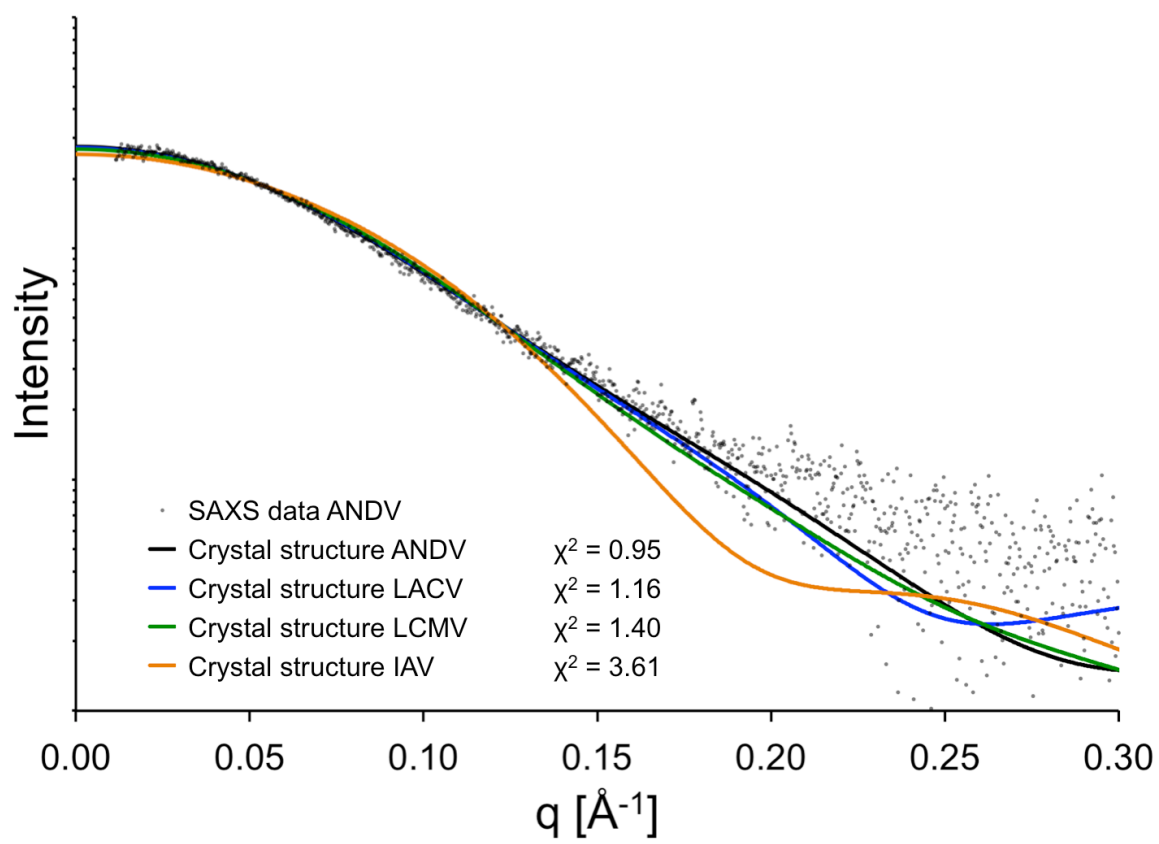

Supplement: S3 Fig — Experimental scattering curves (black dots) are compared with theoretical scattering curves for the ANDV crystal structure (black line), the LACV crystal structure (blue line), the LCMV crystal structure (green line) and the IAV crystal structure (orange line). χ2 –values of fits are given for each structure, curves were calculated with CRYSOL [58]. (PDF) [file ppat.1005635.s003.pdf]

**S4 Figure**

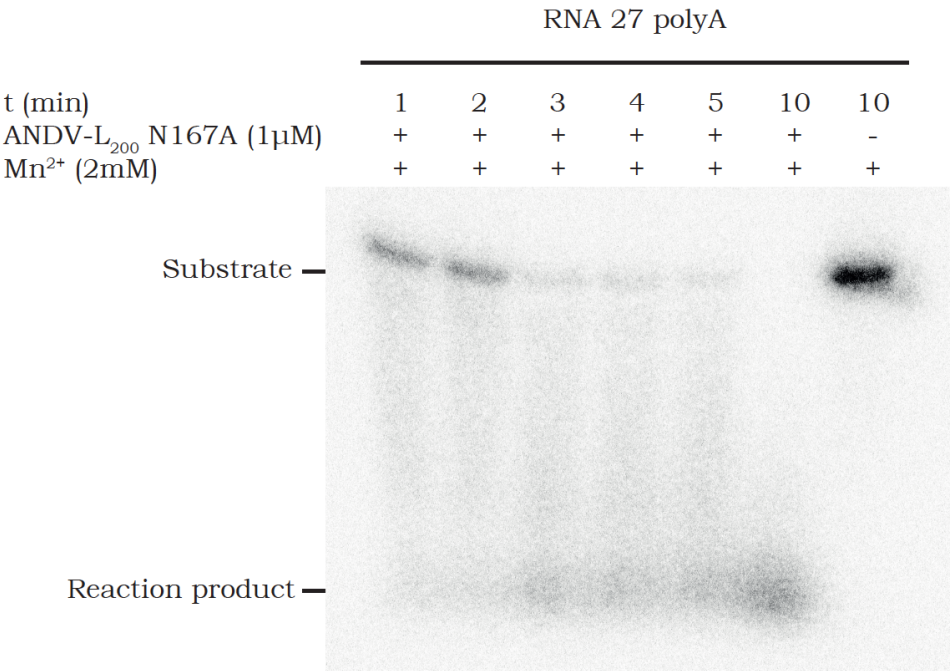

Supplement: S4 Fig — Degradation of unstructured polyA RNA by ANDV L1-200 N167A shows no accumulation of larger intermediate RNA products with specific lengths. (PDF) [file ppat.1005635.s004.pdf]
