# Supplementary material for: Atomic Structure and Biochemical Characterization of an RNA Endonuclease in the N Terminus of Andes Virus L Protein
Source: PLoS Pathog. 2016 Jun 14;12(6):e1005635. doi: 10.1371/journal.ppat.1005635 (PMC4907427; doi:10.1371/journal.ppat.1005635)
Supplement: S1 Table — (PDF) [file ppat.1005635.s005.pdf]

S1 Table. Crystallographic data collection and refinement statistics

	ANDV L_{1-200} K127A anomalous	ANDV L_{1-200} K127A native
Data collection		
Space group	P 42_12	P 42_12
Cell dimensions (Å/°)	a=b=82.9; c=79.3	a=b=82.9; c=79.3
	α=β=γ=90°	α=β=γ=90°
Wavelength (Å)	1.77	0.984
Resolution range (Å)	79.31 – 2.80 (2.87 – 2.80)	79.31 – 2.40 (2.46 – 2.40)
Completeness (%)[1]	99.88 (100)	99.3 (99.6)
Observed reflections	93605 (18108)	84120 (18266)
Unique reflections	13170 (2446)	20577 (4391)
Redundancy[1]	7.1 (7.4)	4.1 (4.2)
R_{merge} (%)[1]	10.9 (126.9)	4.9 (98.0)
Mean I/σ[1]	14.2 (1.8)	19.3 (2.1)
Wilson B-factor	59.74	52.16
Refinement statistics		
R_{work} (%)/R_{free} (%)[2]		20.8/26.2 (38.2/42.5)
Average B-factor (Å^2)		72.4
Protein		72.6
Ligand		74.0
Solvent		67.7
Number of atoms		1672
Protein		1617
Ligand		12
Water molecules		42
RMSD bond lengths		0.01
RMSD bond angles		1.44
Ramachandran favored (%)		93
Ramachandran outliers (%)		0
PDB ID		5HSB

[1] Values in parentheses are for the highest resolution shell.

[2] R_{free} is the cross-validation R factor for 10% of reflections against which the model was not refined.
